# Predictability of drug-induced liver injury by machine learning

**DOI:** 10.1186/s13062-020-0259-4

**Published:** 2020-02-13

**Authors:** Marco Chierici, Margherita Francescatto, Nicole Bussola, Giuseppe Jurman, Cesare Furlanello

**Affiliations:** 1 Fondazione Bruno Kessler, Via Sommarive 18, Trento, 38123 Italy; 2 Department CIBIO, University of Trento, Via Sommarive 9, Trento, 38123 Italy

**Keywords:** Deep learning, DILI, Classification, Microarray, CMap

## Abstract

**Background:**

Drug-induced liver injury (DILI) is a major concern in drug development, as hepatotoxicity may not be apparent at early stages but can lead to life threatening consequences. The ability to predict DILI from in vitro data would be a crucial advantage. In 2018, the Critical Assessment Massive Data Analysis group proposed the CMap Drug Safety challenge focusing on DILI prediction.

**Methods and results:**

The challenge data included Affymetrix GeneChip expression profiles for the two cancer cell lines MCF7 and PC3 treated with 276 drug compounds and empty vehicles. Binary DILI labeling and a recommended train/test split for the development of predictive classification approaches were also provided. We devised three deep learning architectures for DILI prediction on the challenge data and compared them to random forest and multi-layer perceptron classifiers. On a subset of the data and for some of the models we additionally tested several strategies for balancing the two DILI classes and to identify alternative informative train/test splits. All the models were trained with the MAQC data analysis protocol (DAP), *i.e.*, 10x5 cross-validation over the training set. In all the experiments, the classification performance in both cross-validation and external validation gave Matthews correlation coefficient (MCC) values below 0.2. We observed minimal differences between the two cell lines. Notably, deep learning approaches did not give an advantage on the classification performance.

**Discussion:**

We extensively tested multiple machine learning approaches for the DILI classification task obtaining poor to mediocre performance. The results suggest that the CMap expression data on the two cell lines MCF7 and PC3 are not sufficient for accurate DILI label prediction.

**Reviewers:**

This article was reviewed by Maciej Kandula and Paweł P. Labaj.

## Background

Adverse drug reactions (ADRs) are a major threat to the development of novel drugs and their therapeutic use [1, 2]. A particular class of ADRs is drug induced liver injury (DILI), encompassing ADRs that cause liver damage. The liver is the most common target of ADRs, because of its crucial role in the metabolism of endogenous and exogenous compounds [3]. Predictive markers of DILI able to identify susceptible patients would give an enormous advantage to accelerate safe drug development and to prevent severe reactions after approval [4, 5]. DILI poses particular challenges, as pre-clinical testing for side effects in animals does not automatically transfer to clinical trials and then to post-marketing treatment in the population. Indeed, individual susceptibility may arise in patients different from those enrolled in trials, or range from clinically serious to worse as a function of interaction with other factors [6].

A number of groups have developed approaches and strategies to predict DILI from different data types, such as compound chemical structures, gene expression and genetic data. Modelling based on chemical structures and molecular descriptors has been broadly used for DILI prediction (see for example [7–10]). Interestingly, Xu et al. [11] proposed a deep learning (DL) model that achieved 86.9% classification accuracy in external validation after training on a set of 475 samples. Fewer studies have focused on the of use gene expression signatures for ADR or DILI prediction [12–14]. Kohonen and colleagues recently proposed a large-scale data-driven modeling approach to build a predictive toxicogenomics space (PTGS) combining the US Broad Institute Connectivity Map (CMap [15]) and the US National Cancer Institute 60 tumour cell line screen (NCI-60 [16]). Using the PTGS they were able to predict clinical exposure levels raising DILI concerns achieving, in combination with other hepatocellular-based assays, a positive predictive ability of 72–86%.

The Critical Assessment Massive Data Analysis (CAMDA) group proposed in 2018 the CMap Drug Safety Challenge. The challenge task was predicting human clinical DILI from the gene expression responses of two cancer cell lines (MCF7 and PC3) to distinct drug compounds, part of the larger CMap build 02. A recommended split into train (TR) and test (TS) sets and corresponding binary DILI response labels for 276 drug compounds were provided. The dataset presents with a number of technical issues to tackle. The overall number of samples is small, resulting in a limitation for training complex models. The two DILI classes are highly imbalanced, with the largest class including over 70% of the samples: this is potentially an issue, as most machine learning algorithms work better when the classes contain roughly the same number of samples [17]. Finally, the data includes expression of both compound-treated and untreated samples, and these need to be taken into account appropriately. We developed three DL models to predict DILI on the challenge data and compared their accuracy with shallow machine learning models (SL), namely a random forest classifier (RF) and a baseline multi-layer perceptron (MLP). Models combining response to both drug and corresponding vehicles were investigated, as well as strategies for class balancing and identification of alternative informative TR/TS splits. The Matthews correlation coefficient (MCC [18, 19]) was used to assess the performance of our models, as it effectively conveys in a single number the confusion matrix of a classification task, thus making it possible to evaluate classifier performance even in presence of unbalanced classes.

## Results

**Fig. 1:**
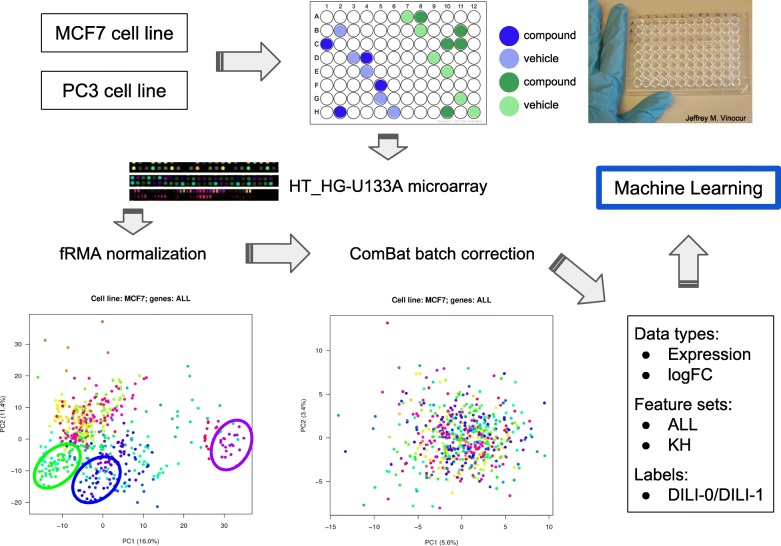
Experimental design scheme and batch correction. The figure represents schematically the data processing approach adopted in the article

Data production and processing layout are outlined in Fig. 1. Briefly, the microarray data for compounds and vehicles was pre-processed, normalized and batch corrected following a standard procedure. Two distinct feature sets were extracted: ALL (including all 12437 genes with detectable expression) compared with KH (the 1234-gene PTGS signature proposed in [14]). All the models were trained on 187 drugs within a standard data analysis protocol (DAP) and validated on 79 different drugs, using as input either the compound expression values or the log-fold change (logFC) of compounds vs. vehicles. All processing steps are detailed in the “Methods” section. Considering our results globally, the general classification performance for the DILI status was poor. MCC values in CV ranged from −0.04 to 0.21, while MCC in validation ranged from −0.16 to 0.11 (details below). These results are comparable with a random labels experiment on the same data. We did not identify a model that performs systematically better than the others, nor important differences in classification performance when considering separately the two cell lines, the different feature sets or the different input types. The results of all experiments performed are collected in Additional file 3.

### Deep Learning

**Fig. 2:**
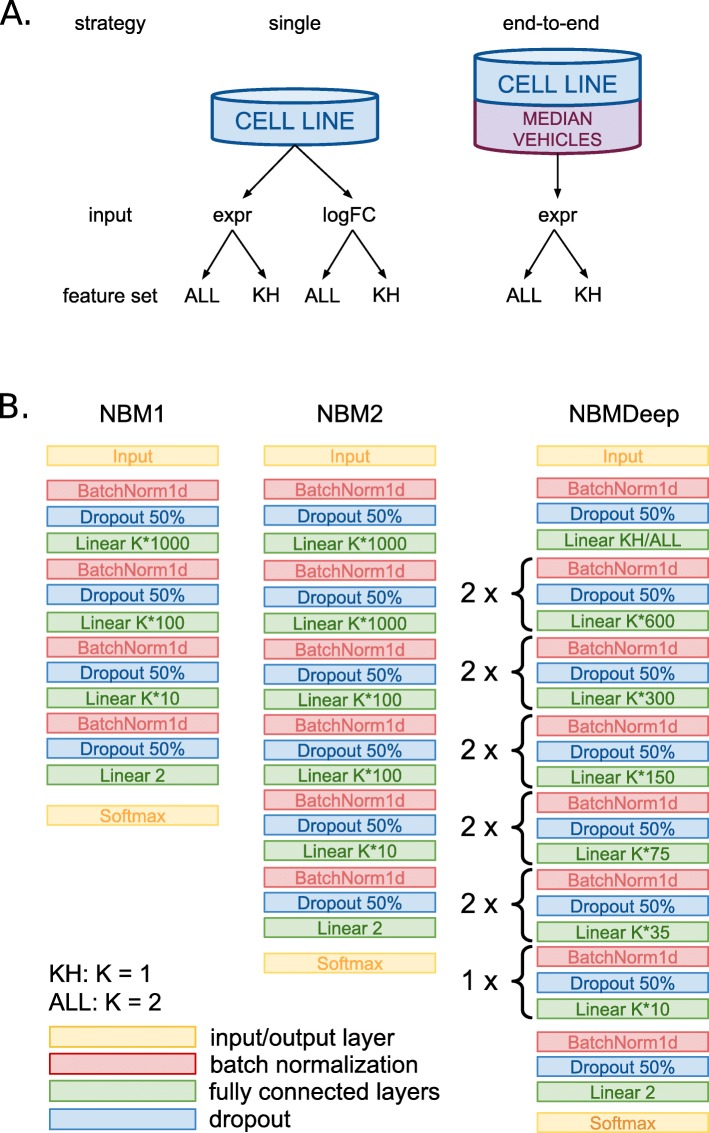
Deep learning analysis strategies and architectures. **a** Strategies used for the analysis. “single” indicates that the logFC values or the expression of each compound were considered as input for the models; “end-to-end” indicates that the expression values of each compound are considered along with its corresponding vehicles. **b** Schematic representation of the DL architectures used for the analysis

We devised three DL architectures of increasing depth, namely NBM1, NBM2, NBMDeep (Fig. 2; see Methods for details), with 4, 6, and 13 hidden layers, respectively. All DL models operated in two modes: “single”, with the logFC values or the expression of each compound as inputs, or “end-to-end”, with the expression values of each compound concatenated with its corresponding vehicles as inputs. Overall, the classification performance was poor independently of the architecture, the DL strategy, and the cell line. In particular, all the DL models performed poorly on the two cell lines (median MCC_cv,MCF7_=MCC_cv,PC3_=0.02; MCC_val,MCF7_=0, MCC_val,PC3_=−0.02), using the two feature sets or input types. The MCC values of the DL “end-to-end” experiments were higher in CV than the “single” experiments (median MCC_cv,end-to-end_=0.09, MCC_cv,single_=0.01; Wilcoxon *p*=0.003), but close to 0 in validation for both strategies. Notably, the NBMDeep architecture performed worse than NBM1 and NBM2, achieving median MCC=0 both in cross-validation and validation for each experiment. Qualitatively, NBM1 performed slightly better than NBM2 in CV (median MCC_cv,NBM1_=0.07, MCC_cv,NBM1_=0.03; *p*=0.31), showing opposite behavior in validation (median MCC_val,NBM1_=−0.06, MCC_val,NBM2_=−0.02; *p*=0.25).

### Shallow machine learning

**Fig. 3:**
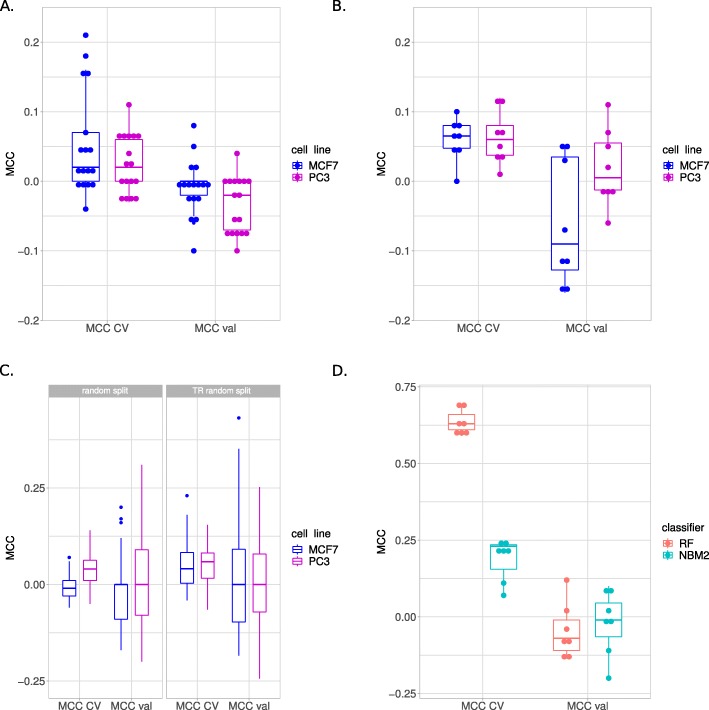
Classification results. **a** Overall DL results. **b** Overall SL results. **c** Random TR/TS splits results. **d** Overall results obtained testing various strategies to balance classes. MCC CV: MCC in CV; MCC val: MCC in validation

To compare the accuracy of the DL models with a SL baseline, we trained two shallow machine learning classifiers, namely a RF and an MLP. Similarly to the behaviour observed for the DL models, the performance of the SL classifiers was poor independently of model, feature set and input type. The average MCC values in CV ranged from 0 to 0.12 for RF and from 0.01 to 0.10 for MLP. The MCC in external validation ranged from −0.12 to 0.07 for RF and from −0.16 to 0.11 for MLP. Overall, the SL experiments displayed comparable CV performance in both cell lines, with slightly worse validation performance in MCF7 than in PC3 (Fig. 3B).

### Random splits

Since the classification performance obtained with both shallow and deep machine learning methods was generally low, we asked whether an alternative TR/TS split could be more informative on the classification task under analysis. To test this hypothesis we randomly split the whole set of 266 samples into 100 random TR/TS pairs containing 75% and 25% of the data respectively. As the classification performance was particularly low in external validation, we performed a similar experiment considering the TR set alone. The results are shown in Fig. 3C. In general, the average classification accuracy over the 100 splits generated from the whole dataset was slightly higher (*p*<0.01) on PC3 (mean MCC_cv_=0.04; mean MCC_val_=0.02) than on MCF7 (mean MCC_cv_=−0.01; mean MCC_val_=−0.03). We concluded that an alternative and more informative TR/TS partition could not be found among the 100 splits. Considering the splits generated from the training set only, the average classification accuracy was marginally better in both PC3 (mean MCC_cv,PC3_=0.05; mean MCC_val,PC3_=0.01) and MCF7 (mean MCC_cv,MCF7_=0.05; mean MCC_val,MCF7_=0.03).

### Class balancing

As shown in Table 1, the two DILI-1 and DILI-0 classes are not represented equally, as over 70% of the samples are DILI-1. To test whether class balancing might be beneficial to improve the classification performance we tested a number of balancing strategies offered by the imbalanced-learn [20] package. The class balancing experiments were performed on the cell line MCF7, with the feature set KH, using expression as input and either RF or NMB2 as classifier. The results are detailed in Table 2 and Fig. 3D. In general, class balancing improved the classification performance in CV without major impact on external validation performance. Notably, for all the balancing strategies tested, RF performs starkly better than NBM2 in CV (average MCC_cv,RF_=0.64 vs. average MCC_cv,NBM2_=0.19). However, performances in validation were again poor (average MCC_val,RF_=−0.05 vs. average MCC_val,NBM2_=−0.02). This suggests that RF is more prone to overfitting the TR set when the least represented class is artificially augmented.

**Table 1:** Number of samples belonging to DILI-0 and DILI-1 classes for TR and TS sets

	DILI-1	DILI-0
TR	120	60
TS	67	19

**Table 2:** Results obtained for RF and NBM2 classifiers using different class balancing strategies

balancing strategy	classifier	MCC _*cv*_	MCC _*val*_
adasyn	RF	0.63 (0.60, 0.66)	**0.12**
oversampled_all	RF	**0.69 (0.65, 0.71)**	-0.13
oversampled_minority	RF	**0.69 (0.65, 0.71)**	-0.13
smote	RF	0.63 (0.60, 0.66)	0.02
smote_svm	RF	0.61 (0.59, 0.65)	-0.09
smote_borderline1	RF	0.61 (0.58, 0.64)	-0.04
smote_borderline2	RF	0.59 (0.55, 0.63)	-0.07
adasyn	NBM2	0.07 (0.03, 0.10)	0.02
oversampled_all	NBM2	**0.24 (0.19, 0.29)**	-0.02
oversampled_minority	NBM2	0.23 (0.19, 0.28)	0.07
smote	NBM2	0.20 (0.15, 0.25)	-0.2
smote_svm	NBM2	**0.24 (0.20, 0.29)**	**0.1**
smote_borderline1	NBM2	0.23 (0.19, 0.29)	-0.11
smote_borderline2	NBM2	0.11 (0.06, 0.16)	-0.01

Boldface indicates the best performance of RF or NBM2 models either in cross validation or in validation

## Discussion

In the context of the CAMDA2018 CMap Drug Safety Challenge we performed an array of machine learning experiments to assess the capability of classifying DILI status from expression data derived from the two cancer cell lines MCF7 and PC3. We built three DL architectures to solve the assigned DILI classification task and compared their performance to two shallow machine learning algorithms (RF and MLP). Overall, we observed very poor classification performance both in CV and in validation, independently on cell line, feature set and classifier. Notably, the NBMDeep architecture performed significantly worse than the two shallower DL architectures, possibly due to a much larger number of parameters to train with limited data. A reduced number of samples is notoriously a limit for the applicability of DL. We investigated the existence of a better TR/TS split by randomly splitting the 266 samples into 100 artificial TR/TS splits containing 75 and 25% of the data. The results on these simulated TR/TS splits did not highlight the presence of a more informative partition of the data. We additionally questioned whether the low MCC values obtained in validation indicate that the TR and TS samples are extracted from two distinct data distributions regardless of normalization. To indirectly test this hypothesis we randomly split the 180 samples of the TR set into 100 artificial TR/TS splits. The results obtained were in line with the random splits on the full dataset. As the two DILI classes were fairly imbalanced we tested two of our classifiers on a subset of the data (MCF7 expression data restricted to the KH feature set) with classes artificially balanced following multiple strategies. The results show a sharp improvement for MCC in CV (9.7 and 7.7 times for the RF and DL classifiers, respectively) with essentially no improvement in external validation, suggesting that the balancing strategies give rise to overfitting. An objective comparison with previous efforts aiming at DILI prediction is challenging, as most studies relied on compound chemical structures and molecular descriptors to assess DILI risk [7–10, 21]. The closest study we can consider for comparison is Kohonen et al. [14] as they also used CMap transcriptomics data for the creation of a DILI prediction score. However, the authors used the full CMap dataset, including ca. 1300 compounds and three cell lines, combined with the NCI-60 cytotoxicity data [16]. As the input is fundamentally much larger and therefore more suitable for training a model, a direct comparison with the classification strategies presented here is difficult to interpret.

## Conclusions

All our experiments point to the major conclusion that the data provided in the context of the CAMDA2018 CMap Drug Safety Challenge do not grant the capability of classifying the DILI status.

## Methods

### Data

The data provided by the CAMDA2018 organizers included microarray expression derived from two cell lines (MCF7 and PC3), either treated with one of 276 chemical compounds or dimethyl sulfoxide (DMSO) vehicle alone, part of the larger Connectivity Map build 02 resource [15]. A spreadsheet containing information to link compound filename identifiers to the corresponding vehicles, the DILI labels for the 276 compounds and the split into TR and test TS sets was also provided (Additional file 1). To complement these information, we downloaded from the CMap project website a sample annotation file (Additional file 2) including information such as chip platform used for the assay, processing batch identifiers, compound CMap names, treatment duration and compound concentration during treatment. Experiments were performed in 96-well plates and a graphical representation of the experimental design is provided in Fig. 1 along with the data pre-processing overview. The original dataset provided by the organizers globally included 1095 CEL files (Table 3). Two distinct Affymetrix chips were used for the expression data assays: HG-U133A and HT_HG-U133A. To avoid potential confounding effects in the analysis, since HG-U133A was used only for a handful of samples, these were removed from the list of input CEL files prior to normalization. Consequently, the starting dataset consisted of a total of 1057 samples, distributed across cell lines as shown in Table 4.

**Table 3:** CEL files available in the original CAMDA2018 Drug Safety challenge dataset

Affymetrix chip	MCF7	PC3
HT_HG-U133A	588	475
HG-U133A	7	25

**Table 4:** Number of samples available after removing CEL files profiled with the HG-U133A chip

category	MCF7	PC3
compound train	180	180
compound test	86	86
vehicle	316	209

Sample numbers are reported according to three categories: samples treated with a compound assigned to the TR test, samples treated with a compound assigned to the TS set and samples treated with DSMO vehicle only

### Microarray data preprocessing

The microarray data was normalized using the fRMA function of the Bioconductor package fRMA [22] with default parameters. Briefly, the function performs background correction according to the robust multi-array average algorithm, quantile normalization and robust weighted average summarization over probesets. Using the Bioconductor annotation package hgu133a.db [23], the expression data was further summarized considering the mean expression value for each gene and gene symbols were used as reference. Since a batch effect related to the actual microarray processing batches was observed, a batch correction adjustment was applied to the normalized expression data using the ComBat function of the Bioconductor package sva [24]. The resulting normalized and batch adjusted data was used as input for the subsequent analyses, either directly in the form of compound expression or as the log_2_-transformed fold change (logFC) between compound and vehicle treated samples. If a given compound was associated to multiple vehicles, their median expression value was considered in the calculation. All data were simultaneously normalized, neglecting the TR/TS partition due to their mutual heterogeneity. We note that part of the vehicles were shared between the TR and the TS set. We considered two feature sets. A first dataset included all the 12437 genes resulting from the processing of the microarray data (named ALL feature set). A second, more compact, consisted of 1234 genes (KH feature set) representing the intersection between ALL and the 1331 genes most associated to the predictive toxicogenomics space defined by Kohonen and colleagues in [14].

### Deep learning architectures

The DL models were trained following two distinct strategies dealing with vehicle expression differently, as sketched in Fig. 2A. In the first strategy (“single”) each cell line was treated independently and either the logFC values or the expression of each compound were considered as input for the models, creating samples of size (1×N), with N=12437 (ALL) or N=1234 (KH). In the second strategy (“end-to-end”), we considered the expression of each compound along with the median of the corresponding vehicles, creating homogeneous samples of size (2×N) for each cell line, with N=12437 (ALL) or N=1234 (KH).

We designed three neural network architectures with increasing depths: NBM1, NBM2, and NMBDeep (Fig. 2B). The NBM1 architecture includes a first layer taking as input the whole set of 12437 (ALL) or 1234 (KH) features, concatenated according to the two strategies. This is followed by two fully connected layers with 1000*K* and 100*K* nodes (with K=2 for ALL and K=1 for KH) and by the output layer. NBM2 was created doubling the 1000K and 100K inner layers of NMB1. NBMDeep is the deepest network, created further expanding the inner layers of NBM2 as detailed in Fig. 2B, obtaining a total of 12 hidden layers.

For each architecture the weights and biases of the fully connected layers were initialized before training with values drawn from the uniform distribution. The rectified linear unit (ReLU) functions [25] were used as activations for all the inner layers while SoftMax was used for the output layer. For the ReLU layers a batch normalization with eps 10^−5^ and momentum 0.1 was applied. The categorical cross-entropy was chosen as loss function, with weights proportional to the class sizes. To avoid overfitting, dropout layers were added with rate 0.5 after each of the inner layers. The networks were trained over 1000 (NBM1, NBM2) or 5000 (NBMDeep) epochs, using minibatches of 60 samples.

#### Parameter tuning

The optimizer type and the learning rate (LR) of the networks were selected among the alternatives described below by training NBM1 over 1000 epochs on 70% of the training set (randomly chosen) and evaluating the performance on the left-out 30% portion. With the stochastic gradient descent (SGD) optimizer, the net was trained with LR∈[10^−2^,5×10^−3^,2×10^−3^,10^−3^]. Using Adam optimizer, the net was trained with LR∈[10^−7^,10^−6^,5×10^−6^,7×10^−6^,8×10^−6^,9×10^−6^,10^−5^,10^−4^,5×10^−4^,10^−3^], as Adam requires smaller LR with respect to SGD [26]. We compared the training and validation performance and losses of the network using the two optimizers. As detailed in the “Results” sections, the performances were generally poor without strong dependence on the parameters. We decided to use Adam as optimizer with LR=1×10^−5^ as it was giving slightly better performance (not shown).

### Shallow machine learning

We considered a basic MLP and a RF as baseline machine learning strategies to compare our DL models to. MLP consisted of three fully connected hidden layers with 30 nodes each, and an input layer with 12437 or 1234 nodes for ALL and KH feature sets, respectively. All activations were ReLU functions [25], with neither dropout nor batch normalization. As optimizer we used Adam [26] with the number of iterations bounded at 200. RF was initialized with 500 trees and the Gini impurity as criterion to evaluate the quality of a split.

### Random splits

We randomly split either the whole dataset or the original TR set into new TR/TS pairs, containing 75% and 25% of the data respectively with balanced classes, 100 times. Since previous experiments showed fundamentally homogeneous results across classifiers and feature sets, the “random split” experiments were performed using the RF classifier and the ALL feature set for both cell lines.

### Class balancing

Since the TR and TS classes were unbalanced (including about two thirds vs. one third of the initial data respectively) three oversampling strategies were considered for balancing, as follows:

- naïve random over-sampling, i.e. resampling either both classes (*all*) or the minority class only (*minority*);
- synthetic minority oversampling technique (SMOTE, [27]) and variants *borderline1*, *borderline2*, *svm* [28, 29];
- adaptive synthetic sampling approach for imbalanced learning (ADASYN, [30]).

Oversampling was performed using imbalanced-learn v0.3.3 Python package [20]. The experiments were performed on the cell line MCF7, on the feature set KH, using expression as input and either RF or NMBDeep as classifier.

### Predictive modeling strategy

All shallow and DL models (including class balancing experiments) were trained within the DAP previously developed by FBK within the MAQC-II and SEQC challenges [31, 32], the U.S. FDA initiatives for reproducibility of biomarkers. Briefly, our DAP uses a 10×5−fold stratified CV on TR to get a ranked feature list and a set of classification metrics [33], including the MCC. Data were rescaled in the interval [−1,1] (for shallow learning) or centered and scaled to unit variance (for DL) before undergoing classification: rescaling parameters from TR were used for rescaling both TR and TS subsets, so to avoid information leakage. The DL models were run in the DAP without feature selection, which was enabled for MLP and RF.

### Computational details

The NBM1, NBM2 and NBMDeep architectures were implemented in PyTorch v0.40 [34]. The MLP network and the RF models were implemented in scikit-learn v0.19.1 [35]. The whole DAP was written in Python. All DL computations were run on either a Microsoft Azure platform with 4x NVIDIA Tesla K80 GPU cards or on a Linux workstation with 2x NVIDIA GeForce GTX 1080 cards. Shallow learning models were run on the FBK KORE high-performance computing Linux cluster. All plots were produced using the ggplot2 R package [36]. Comparisons between conditions of interest were assessed by Wilcoxon test using the wilcox.test R function.

## Reviewers’ comments

### Reviewer’s report 1

Maciej Kandula

**Reviewer comment:** The manuscript by Marco Chierici *et al* investigate the application of machine learning models on the CMap dataset to predicting drug-induced liver injury (DILI). Specifically, the challenge involves predicting human clinical DILI from the gene expression responses of two cancer cell lines. Authors perform a review of topical and state-of-the-art literature, discussing some recent works that achieved high predictive performance with regard to DILI and using gene expression (Kohonen et al, 2017). Kohonen *et al* used, among other information, the same two cell lines from the cMap dataset: MCF7 and PC3, that Chierici *et al* analyse in this work. Authors describe their analysis plan in detail, emphasizing the importance of comprehensive evaluation. They compare Deep Learning models’ performance with multiple shallow learning methods in a cross-validation approach. The architectures of the deep learning models proposed are clearly depicted in a figure. Authors do justify their choices with regard to hyperparameter selection. The selection process is discussed briefly but by no means exhaustively, and some other choices could potentially benefit the overall performance. They are aware of the potential limitations of the analysed dataset, like small sample size and imbalanced label distribution and develop strategies to overcome these issues. The poor performance of the evaluated algorithms is unfortunate. Predicting DILI from expression data seems, however, to be very difficult in general. Given the above, I do have some minor concerns that the authors should address before publishing their work: (1) Kohonen *et al* also used the cMap gene expression data (MCF7 and PC3) and they achieved very good predictive performance. You do use their dimensionality reduction / feature selection strategy but your methods still perform poorly. Why so? How does your study compare to theirs? It seems that you only work on a small selection of compounds from a larger dataset but this is not clear from the text. ***Author’s response:*** We have indeed discussed in the manuscript (“Discussion” section) these issues. We respectfully note that a direct comparison of our results with the performance of Kohonen et al. models is not directly applicable due to significant differences in data and goals of the analyses in the two works. First, Kohonen et al. target is prediction of “clinical exposure levels raising DILI concerns”. This is substantially different from prediction of DILI labels, as defined by the DILIrank database in our paper, which is a focused resource used for reproducibility with other teams in the context of the CAMDA challenge, rather than the cMAP build 2 full dataset employed by Kohonen et al. The manuscript indeed reports that the 276 compounds used in our work are a subset of the larger cMAP build 2 resource (“Methods” section).

**Reviewer comment:** (2) You are clear that you do not think these expression data are informative and can be used for DILI prediction. Is there something that could be added or improved that could help to improve the performance of your models? Or could you suggest other models that could potentially work better? It seems that using more data could improve the performance. ***Author’s response:*** The aim of the manuscript is to assess the predictability of DILI from gene expression data only (see also Background, Discussion). We cite previous work (e.g. Xu et al. 2015, ref. 11 in the manuscript) that found good classification performance achieved using relevant molecular features for classification. The integration of molecular features and expression data could indeed improve classification performance; in recent work from our lab (Fabbri L., unpublished 2019) two methods (Random Forests and attention-based deep neural networks) applied to multiple molecular descriptors and their combinations were used to predict DILI from the complete DILIrank database. In particular, the integration of structural features with biological information (e.g., chemical-protein interaction network properties from the STITCH database) improved over published work (Hong, Thakkar et al, 2017: ref. 9 in the manuscript). We have added the reference to Fabbri 2019 in the same (“Discussion”) section.

**Reviewer comment:** (3) The hyperparameter selection is discussed briefly and it is clear that not many parameters were actually considered. Do you think any other choices could potentially benefit the overall performance? ***Author’s response:*** The hyperparameter grid may certainly be expanded (*e.g.* varying the number of layers/neurons in the DL architecture). Given the extremely poor results of all the models we tested, we do not expect that markedly better performance can be achieved by simply expanding the search grid. We therefore decided to adopt a simple scheme.

**Reviewer comment:** (4) Authors provide a link to an online repository with code used for this analysis but I was unable to log into it. Please have a look into it. ***Author’s response:*** The link to our repository has been fixed.

### Reviewer’s report 2

Paweł P. Labaj

**Reviewer comment:** The manuscript by Chierici *et al* presents an extensive study of the limits of machine learning in the face of biomedical data sets limited by sample size and hidden signals. They dive deep into the international data analysis challenge of predicting drug induced liver injury (DILI) from gene expression profiles from drug compound cell-line assays, which was assembled by the US FDA in the framework of the Critical Assessment of Massive Data Analysis conference (CAMDA, www.camda.info). Specifically, the team follows best practise through a data analysis plan established by the US FDA MAQC2 consortium, including 10x5 cross-validation. The authors examine 3 deep learning architectures in comparison to two less complex classification approaches. In the thorough comparison to randomised labels and in independent external validation, it turns out that none of the approaches works very well. Rather than stop at this point, the authors then dissect this issue further. They attempt to rebalance the highly skewed sample labels, which interestingly leads to overfitting of the methods of greater complexity, indicating that in-build regularisation does not save them from overfitting the augmented data. In the end, it seems that the attempt to prepare a cleaner, smaller data set with thoroughly curated DILI labels could not overcome the inherent limitations of smaller sample size, unbalanced label categories, and the conceptual distance of gene expression profiles from cell line assays to the eventual regulatory DILI classification of a drug. In comparison, the Kohonen paper from 2017 could find better performance in an about 6x larger dataset, also linking it to toxicological data. Still, I much recommend this paper for publication because it is one of a small number of manuscripts that report a negative result ’and’ derive interesting insights from a thorough dissection of the analysis. I think the manuscript is ready for publication in its present form. ***Author’s response:*** We thank the reviewer for the critical evaluation of our work and the positive feedback.

## Supplementary information

**Additional file 1** CAMDA filenames. The spreadsheet provided by the CAMDA2018 conference organizers. It contains the names of the files included in the CMap Drug Safety challenge, as well as the binary DILI label for each compound and the split into TR and TS sets.

**Additional file 2** CMap sample annotation file. Sample annotation file downloaded from the CMap project website (https://portals.broadinstitute.org/cmap/). The file includes the following information for all samples available in CMap build2: batch_id, cmap_name, INN, concentration, duration, cell, array, perturbation_scan_id, vehicle_scan_id, scanner, vehicle, vendor, catalog_number, catalog_name.

**Additional file 3** All classification results. The file includes performance tables for each classification experiment run in the manuscript.

## Data Availability

The datasets supporting the conclusions of this article are available in the CAMDA2018-cmap-DILI repository, https://gitlab.fbk.eu/toxpred/CAMDA2018-cmap-DILI.

## Abbreviations

ADASYN: Adaptive synthetic sampling approach for imbalanced learning
ADR: Adverse drug reaction
ALL: Feature set including all genes for which expression is detected
CAMDA: Critical Assessment Massive Data Analysis
CMap: Connectivity Map
DAP: Data analysis protocol
DILI: Drug induced liver injury
DL: Deep learning
DMSO: Dimethyl sulfoxide
KH: Feature set including only expressed genes belonging to the PTGS signature
logFC: Log fold change
LR: Learning rate
MCC: Matthews correlation coefficient
MLP: Multi-layer perceptron
NCI-60: National Cancer Institute 60 tumour cell line screen
PTGS: Predictive toxicogenomics space
ReLU: Rectified linear unit
RF: Random forest
SGD: Stochastic gradient descent
SL: Shallow machine learning
SMOTE: Synthetic minority oversampling technique
TR: Train
TS: Test
